# Assessing quality, reliability and accuracy of polycystic ovary syndrome‐related content on TikTok: A video‐based cross‐sectional analysis

**DOI:** 10.1002/ijgo.70007

**Published:** 2025-02-10

**Authors:** Gaetano Riemma, Raffaela Maria Carotenuto, Camilla Casolari, Carlo Ronsini, Luigi Della Corte, Marco La Verde, Vittorio Unfer, Pasquale De Franciscis, Antonio Simone Laganà, Andrea Etrusco

**Affiliations:** ^1^ Obstetrics and Gynecology Unit, Department of Woman, Child and General and Specialized Surgery University of Campania “Luigi Vanvitelli” Naples Italy; ^2^ Department of Neuroscience, Reproductive Sciences and Dentistry, School of Medicine University of Naples Federico II Naples Italy; ^3^ The Experts Group on Inositol in Basic and Clinical Research, and on PCOS (EGOI‐PCOS) Rome Italy; ^4^ Department of Gynecology UniCamillus‐Saint Camillus International University of Health Sciences Rome Italy; ^5^ Unit of Obstetrics and Gynecology, “Paolo Giaccone” Hospital, Department of Health Promotion, Mother and Child Care, Internal Medicine and Medical Specialties (PROMISE) University of Palermo Palermo Italy

**Keywords:** adolescents, misinformation, PCOS, quality assessment, social network, TikTok

## Abstract

**Objective:**

Social networks share medical content with no peer‐review or fact‐checking. In the present study we aimed to assess the quality, reliability, and level of misinformation in TikTok videos about polycystic ovary syndrome (PCOS).

**Methods:**

We performed a cross‐sectional analysis of TikTok videos retrieved using “PCOS” as the search term and analyzed using patient education materials assessment tool for audio‐visual content (PEMAT A/V), modified DISCERN (mDISCERN), global quality scale (GQS), video information and quality index (VIQI) and misinformation assessment were employed.

**Results:**

A total of 180 videos were included. Most videos were partially accurate (containing 25%–50% of false information) or uninformative (more than 50%) (56.7% and 6.6%, respectively) with a significantly higher proportion of inaccurate or uninformative videos from PCOS‐patients than healthcare professionals (14.4% vs. 0%; *P* < 0.001) as well as for partially accurate videos (78.4% vs. 37.5%; *P* < 0.001). PEMAT A/V scores for understandability and actionability were 50% (interquartile range [IQR]: 33%–58%) and 25% (IQR: 25%–50%), respectively with significantly higher understandability for healthcare professionals (54% [IQR: 42%–71%] vs. 33% [IQR: 25%–50%], *P* < 0.001). Median mDISCERN was 2 (IQR: 1–3) (low degree of reliability), with videos by healthcare professionals scoring significantly higher than those by patients (2 [IQR: 2–3] vs. 1 [IQR: 0–2]; *P* = 0.001).

Intermediate‐low overall video quality was reported in VIQI with median score of 12 (IQR: 10–15) and significantly lower scores for patients (9 [IQR: 5–12] vs. 13 [IQR: 12–17]; *P* < 0.001). Similarly, median GQS score was overall intermediate for degree of usefulness (median 3 [IQR: 2–4]), but patient‐created videos were of significantly lower quality (median 2 [IQR: 2–3] vs. 4 [IQR: 3–4]; *P* < 0.001).

**Conclusion:**

PCOS‐related videos on TikTok were mostly misinformative and of low quality and reliability. Healthcare professionals' videos were more informative with had higher quality compared to patient‐created content. Identifying and addressing low‐quality content is crucial for guiding future public health initiatives and improving the dissemination of trustworthy medical information on social networks.

## Synopsis

PCOS‐related TikTok videos appear limited in trustworthiness, correctness, understandability and actionability. More reliable and accurate content is needed to improve standards of health education on the social network.

## 1 INTRODUCTION

Polycystic ovary syndrome (PCOS) is one of the most prevalent endocrine and metabolic disorders, affecting approximately 10%–13% of women of reproductive age.^1^ It is characterized and diagnosed, according to the 2023 international evidence‐based guidelines for PCOS, using the revised Rotterdam criteria, which requires the presence of at least two out of three criteria: (1) clinical or biochemical signs of hyperandrogenism, (2) ovulatory dysfunction, and (3) polycystic ovarian morphology observed via ultrasound, with the option of using anti‐Müllerian hormone (AMH) levels as an alternative to ultrasound.^2^ Clinically, irregular menstrual cycles due to ovulatory dysfunction and hyperandrogenism (e.g., hirsutism, acne, seborrhea, androgenic) alopecia are present in younger patients.^3^, ^4^ Such conditions can severely affect patient's quality of life, yet many women feel dissatisfied with the information provided by healthcare professionals during diagnosis and treatment.^5^ Social networks serve as a primary resource for women, particularly adolescents, seeking information on reproductive health issues such as PCOS.^6^ These platforms provide access to details about symptoms, treatment options, and long‐term health complications.^7^ Unlike traditional in‐person counseling, many women prefer the internet due to its privacy, accessibility, convenience, and the autonomy it offers in managing the information they receive.^8^ Additionally, online social platforms enable women to exchange advice and foster a sense of community, helping them cope with the challenges of living with PCOS.^6^ However, research on the accuracy and quality of online information remains limited.

TikTok has recently emerged as one of the most popular video‐based social media platforms for disseminating and acquiring health‐related information, boasting over one billion active users worldwide each month.^9^ Previous studies have examined the quality and nature of information regarding PCOS on other social networks, such as YouTube.^10^ However, the characteristics of PCOS‐related content on TikTok remain largely unexplored, particularly with respect to its quality and reliability. The platform's model, which does not require verification of content creators' credibility or expertise, raises concerns about the potential spread of inaccurate or amateurish information.^11^ This issue is exacerbated by the absence of a peer‐review process for content uploaded to TikTok, allowing users to post media at their discretion, without checks for accuracy.^12^

Research has shown that misinformation on social media can negatively impact health behaviors, leading to delayed diagnoses, improper treatments, and increased anxiety among patients.^7^, ^11^, ^13^, ^14^, ^15^, ^16^ Individuals with PCOS may come across videos promoting unproven treatments or dietary changes that lack scientific backing, potentially causing harm or confusion.^15^, ^16^ Moreover, the content shared on these platforms often lacks context or fails to provide balanced information, contributing to the spread of myths and misconceptions.^17^

Healthcare professionals and government organizations have voiced concerns over the quality and reliability of health information available on social media platforms. This is especially problematic given the prevalence of shared personal opinions and anecdotal experiences, which may contribute to misinformation.^18^ These concerns are even more significant in relation to endocrine diseases, such as PCOS, which mainly affect young women and are particularly prone to misunderstanding and misinformation.^10^

Accordingly, several studies have already addressed the limitations of TikTok content regarding health issues. In particular, multiple topics regarding women's healthcare, including the use of outpatient hysteroscopy,^19^ pelvic floor dysfunctions,^17^ body image disorders,^20^ menstrual cycle,^21^ uterine malformations,^22^ infertility, pregnancy,^23^ and medical abortion,^23^ were investigated, with all studies reporting poor quality and misinformative content.

Misinformation influences young women beyond personal preferences and impacts larger public health and educational systems, promoting negative behaviors, postponing medical interventions, and stigmatizing real health issues, especially in case of body image or reproductive health.^24^, ^25^ Because of avoidable problems, this jeopardizes personal well‐being and strains healthcare systems.^26^

Therefore, the aim of the present study was to analyze PCOS‐related content on TikTok, evaluating its quality, reliability, and the prevalence of misinformation. By identifying discrepancies between content created by healthcare professionals and patients, this research seeks to highlight the risks posed by unverified health information on social media and provide practical recommendations for improving online health education. Addressing these issues is critical to ensuring that individuals with PCOS receive accurate, trustworthy information that supports their health and well‐being.

## 2 MATERIALS AND METHODS

### 2.1 Video selection and data extraction

On April 30, 2024, a search was executed on TikTok using the keyword “PCOS.” A new account was created specifically for this study to minimize the impact of personalized content generated by the platform's algorithm. The search results were generated using the platform's default algorithm settings, without any modifications, to ensure an unbiased preliminary selection with retained validity. Videos retrieved using the specified keyword were subsequently reviewed according to pre‐established inclusion and exclusion criteria. To be included, videos had to focus primarily on PCOS and either be presented in English or contain no audio. Videos were excluded if they were in languages other than English, unrelated to PCOS, or duplicate content. All relevant data were manually extracted and recorded in a standardized spreadsheet. The collected data encompassed the video's title, duration, description, upload date, hashtags used, geographical location, and engagement metrics such as the number of likes, shares, views, and comments. Additional documentation was made of content creators' follower count, country of origin, total number of videos posted, and the cumulative number of likes received across all content. The primary subject or narrator of the video was classified as either a healthcare professional (including physicians, nurses, midwives, and medical students) or a PCOS‐diagnosed patient.

The evaluation of video content was carried out by a team consisting of one senior gynecologist (GR) specializing in PCOS and reproductive endocrinology, and two Obstetrics and Gynecology residents (RMC and CC). All team members were part of the “Department of Woman, Child, and General and Specialized Surgery” at the University of Campania “Luigi Vanvitelli,” Naples, Italy.

To ensure objectivity, each team member independently reviewed and scored the videos. Any discrepancies in the evaluations were resolved by a further review conducted by another gynecologist (AE) from the Department of Health Promotion, Mother and Child Care, Internal Medicine and Medical Specialties (PROMISE), University of Palermo, Palermo, Italy. This process facilitated the establishment of a consensus on the content evaluations.

### 2.2 Assessment of misinformation, reliability, quality, and accuracy

The videos were assessed using standardized tools. The interpretability and practicality of the content, in order to assess the educational value of health videos, were evaluated through the patient education materials assessment tool for audiovisual content (PEMAT A/V).^27^ This tool comprises 17 items that assess understandability (items 1–13) and actionability (items 14–17). Three responses (agree = 1, disagree = 0, and not available = NA) were allowed. The overall score was displayed as a percentage that was calculated by dividing the number of items that were rated as agree or disagree by the amount of all points, with higher percentages reflecting more understandable and actionable content.

To assess the reliability and credibility of the video content (whether videos provide trustworthy and accurate content to viewers), the modified DISCERN (mDISCERN) scale was employed. Originally developed by Charnock et al.,^28^ the scale consists of five questions. Each affirmative response contributes one point toward a total score of five. The questions are: (1) Are the aims clear and achieved? (2) Are reliable sources of information used (e.g., citation of a publication or identification of the speaker as a specialist)? (3) Is the information presented balanced and unbiased? (4) Are supplementary sources of information recommended for the benefit of the patient? (5) Are areas of uncertainty acknowledged? A higher mDISCERN score reflects greater reliability, with scores of three or higher indicating a high degree of reliability.^28^

The global quality scale (GQS) was used to evaluate the overall quality of the videos from the perspective of a typical viewer.^29^ Originally designed to assess the utility, structure, and accessibility of web‐based resources, the GQS assigns ratings on a scale from one to five, with higher scores indicating higher‐quality content. Videos with a GQS rating of three or above were deemed to provide high‐quality health information.^29^

Additionally, the video information and quality index (VIQI) was used to assess the educational quality and substance of the videos in terms of technical characteristics.^13^ The VIQI is a five‐point rating system with four criteria: video quality, information flow, clarity, and alignment between the video's title and content.^13^

Collectively, using these tools ensured a comprehensive and balanced assessment of the video content. Moreover, each video underwent a fact‐checking process to identify and address any misinformation. This process used established PCOS guidelines and existing literature.^30^, ^31^, ^32^ Videos were categorized as “inaccurate or misinformative” if they contained more than 50% false information, “partially accurate” if they contained 25%–50% false information, and “accurate and evidence‐based” if they contained <25% misinformation.

### 2.3 Statistical analysis

Normality in the data was assessed using the Shapiro–Wilk test. Non‐normally distributed data were summarized using median and interquartile ranges (IQR), while frequency and percentages were used for count data. As non‐parametric data were retrieved, the Kruskal‐Wallis test was used to compare groups, with Dunn's test applied for non‐normally distributed variables, to test the hypothesis that variables from healthcare professionals' content differed from patient‐created videos. Statistical significance was set at a *P* value of <0.05. Relationships between non‐normal variables were evaluated using Spearman's correlation analysis. All statistical analyses were performed using STATA 14.1 (StataCorp LLC, College Station, Texas, USA).

### 2.4 Ethical approval

Institutional Review Board approval was not required, as the study did not involve human subjects or interventions. All information included in the analysis was publicly available on TikTok.

## 3 RESULTS

### 3.1 Videographic characteristics

**FIGURE 1:**
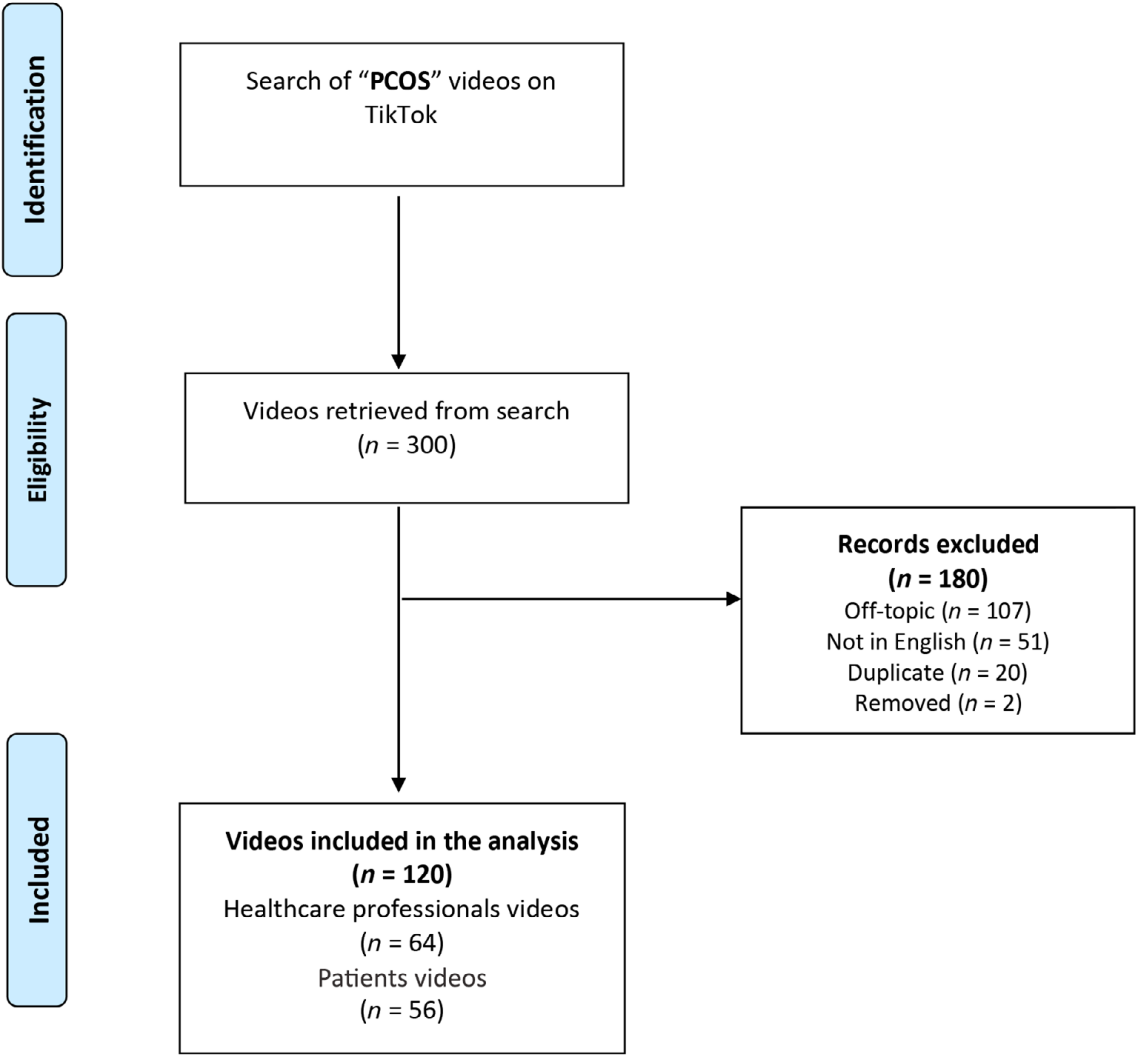
Flow chart depicting the selection process of TikTok videos.

Of the 300 videos reviewed, 120 were deemed suitable for analysis (Table 1). A total of 180 videos were excluded: 107 were off‐topic, 51 were recorded in a language other than English, two were removed from the platform, and 20 were identified as duplicates (Figure 1).

**TABLE 1:** Videographic characteristics of TikTok videos regarding PCOS included in the analysis.

Video characteristics			Healthcare professionals (64)	Patients (56)	*P* value
Length (s)	Median (IQR) Range	53	54	30	0.005.
		(7–269)	(7–269)	(6.5–237)	
		6–302	6–302	1–528	
Views	Median (IQR) Range	42 100	28 800	55 400	0.727
		(1182–1 700 000)	(1182–1 700 000)	(1108–1 900 000)	
		1108–1 900 000	1182–1 700 000	1108–1 900 000	
Comments	Median (IQR) Range	110	77	339	0.012
		(6–1723)	(0–959)	(8–6146)	
		0–9387	0–1157	6–9387	
Likes	Median (IQR) Range	3233	1369	17 576	0.001
		(67–152 949)	(67–100 000)	(106.5–305853.5)	
		55–429 007	62–138 061	55–429 007	
Shares	Median (IQR) Range	11 800	29 600	4580.5	0.001
		(421.5–227 050)	(1517–301 500)	(363–54 100)	
		307–378 000	1277–378 000	307–102 900	
Bookmarks	Median (IQR) Range	277	202	1028.5	0.002
		(7–26 800)	(8–17 600)	(2–60 600)	
		1–94 400	7–91 019	1–94 400	
Author likes	Median (IQR) Range	73 800	105 000	58 550	0.163
		(2256–1 694 550)	(1991–2 500 000)	(2521–1 870 000)	
		995–3 300 000	995–3 330 000	1232–2 690 000	

Abbreviations: IQR, interquartile range; PCOS, polycystic ovary syndrome.

The median duration of the videos was 53 s (interquartile range [IQR]: 7–269 s). The median number of views was 42 100 (IQR: 1182–1 700 000; range: 1108–1 900 000). Table 1 provides detailed statistics on the median numbers of comments, likes, shares, followers, and total likes.

Out of the total videos analyzed, 64 (53.3%) were produced by healthcare professionals, while 56 (46.7%) were created or narrated by patients. All the videos were directed toward a patient‐centered audience. Notably, videos created by healthcare providers were longer and attracted a significantly higher number of shares compared to those produced by patients and followers, while those created by PCOS patients had significantly more view, comments, likes and bookmarks (Table 1).

Table 2 outlines the general characteristics of the analyzed videos. Regarding content categories, healthcare professionals were more likely to present educational material on medical topics, while patients predominantly shared personal narratives and complaints (Table 2; *P* < 0.001). Additionally, the tone of the videos produced by healthcare professionals tended to be more neutral (informative and respectful, without triggering anxiety or being too compliant) compared to those made by patients (Table 2; *P* < 0.001).

**TABLE 2:** Overall characteristics and misinformation assessment of included videos.

Overall		Healthcare professionals (64)	Patients (56)	*P* value
Video category, *N* (%)				
Entertainment	6 (5)	0 (0)	6 (10.7)	<0.001
Medical education	72 (60)	64 (100)	8 (14.3)	
Patient's complaint	18 (15)	0 (0)	18 (32.1)	
Storytelling	24 (20)	0 (0)	24 (42.9)	
Tone of the video, *N* (%)				
Positive	12 (10)	8 (12.5)	4 (7.2)	<0.001
Negative	30 (25)	4 (6.3)	26 (46.4)	
Neutral	78 (65)	52 (81.2)	26 (46.4)	
Purpose, *N* (%)				
Storytelling	32 (26.7)	2 (31.2)	30 (53.6)	<0.001
To educate or inform	82 (68.3)	56 (87.5)	26 (46.4)	
To advertise patients	6 (5)	6 (9.4)	0	
Misinformation assessment, *N* (%)				
Accurate and evidence‐based	44 (36.7)	40 (62.5)	4 (7.2)	<0.001
Partially accurate	68 (56.7)	24 (37.5)	44 (78.4)	
Inaccurate or uninformative	8 (6.6)	0 (0)	8 (14.4)	
Does it describe alternatives to HT?, *N* (%)				
No	86 (71.3)	40 (62.5)	46 (82.1)	0.017
Yes	34 (28.7)	24 (37.5)	10 (17.9)	
Does it encourage patients to avoid HT?, *N* (%)				
No	120 (100)	64 (100)	56 (100)	0.999
Yes	0 (0)	0 (0)	0 (0)	
Does it encourage patients to distrust their gynecologist?, *N* (%)				
No	116 (96.7)	60 (93.7)	56 (100)	0.122
Yes	4 (3.3)	4 (6.3)	0 (0)	
Does it increase anxiety/concern about PCOS?, *N* (%)				
No	88 (73.3)	60 (93.7)	28 (50)	<0.001
Yes	32 (26.7)	4 (6.3)	28 (50)	

Patients were significantly less likely than healthcare professionals to describe other approaches (e.g., physical exercise, inositol or metformin) rather than hormone therapy (17.9% vs. 37.5%; *P* = 0.017), but neither patients nor healthcare professionals encouraged women to avoid hormone treatment (0% vs. 0%; *P* = 0.999). The level of distrust toward gynecologists was extremely low in both video categories (6.3% vs. 0%; *P* = 0.122). Furthermore, patient‐generated videos featured a significantly higher occurrence of anxiety‐related content in comparison to healthcare professionals' videos (50.0% vs. 6.3%; *P* < 0.001) (Table 2).

### 3.2 Misinformation evaluation

Misinformation was assessed by evaluating inaccurate claims about etiology, promotion of unproven treatments, omission of critical information about risks and complications, erroneous dietary advice or claims about fertility. A substantial portion of the videos were considered either partially accurate or lacking in informative value, with 56.7% classified as partially accurate (with 25%–50% of false information) and 6.6% deemed uninformative (more than 50% of false information), accounting for 63.3% of the evaluated material. When analyzed based on the author of the video, a significant distinction emerged between healthcare professionals and patients. A much higher proportion of inaccurate or uninformative videos originated from patients (14.4% vs. 0%; *P* < 0.001) as well as for partially accurate videos (78.4% vs. 37.5%; *P* < 0.001) (Table 2).

### 3.3 Quality evaluation

#### PEMAT A/V

**FIGURE 2:**
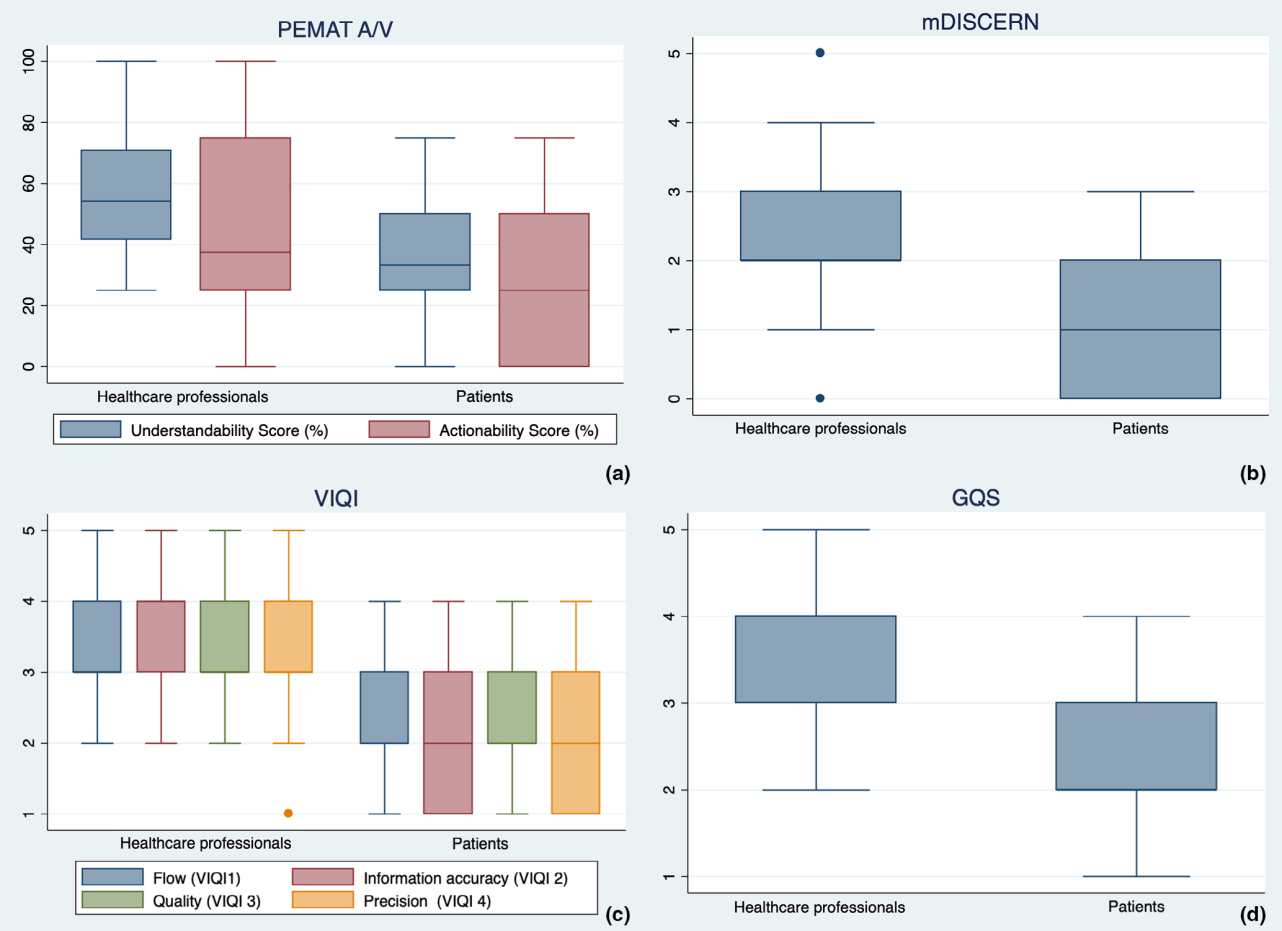
Boxplots (showing median, interquartile, maximum and minimum ranges, and outliers) for (a) PEMAT A/V, (b) mDISCERN, (c) VIQI, and (d) GQS in healthcare workers and patients.

The overall median scores for PEMAT A/V regarding understandability and actionability were 50% (IQR: 33%–58%) and 25% (IQR: 25%–50%), respectively. When examined according to the role of the author of the video, videos produced by healthcare professionals had a significantly higher median score for understandability compared to those made by patients (54% [IQR: 42%–71%] vs. 33% [IQR: 25%–50%], *P* < 0.001). The median actionability score was similar for both groups (37.5% [IQR: 25%–75%] vs. 25% [IQR: 0%–50%], *P* = 0.109) (Table 3; Figure 2a).

**TABLE 3:** Quality assessment according to PEMAT A/V, mDISCERN, VIQI and GQS.

Video characteristics			Healthcare professionals (64)	Patients (56)	*P* value
PEMAT A/V					
Understandability %	Median (IQR) Range	50	54	33	0.001
		(33–58)	(42–71)	(25–50)	
		0–100	25–100	0–75	
Actionability %	Median (IQR) Range	25	37.5	25	0.109
		(25–50)	(25–75)	(0–50)	
		0–100	0–100	0–75	
mDISCERN	Median (IQR) Range	2	2	1	0.003
		(1–3)	(2, 3)	(0–2)	
		0–5	0–5	0–3	
VIQI					
Flow (VIQI1)	Median (IQR) Range	3	3	2	<0.001
		(2–4)	(3, 4)	(2, 3)	
		1–5	2–5	1–4	
Information accuracy (VIQI 2)	Median (IQR) Range	3	4	2	<0.001
		(3, 4)	(3, 4)	(1–3)	
		1–5	2–5	1–4	
Quality (VIQI 3)	Median (IQR) Range	3	3	2	<0.001
		(2–4)	(3, 4)	(2, 3)	
		1–5	2–5	1–4	
Precision (VIQI 4)	Median (IQR) Range	3	3	2	<0.001
		(2–4)	(3, 4)	(1–3)	
		1–5	1–5	1–4	
VIQI score (total)	Median (IQR) Range	12	13	9	<0.001
		(10–15)	(12–17)	(5–12)	
		4–20	8–20	4–15	
GQS	Median (IQR) Range	3	4	2	<0.001
		(2–4)	(3, 4)	(2, 3)	
		1–5	2–5	1–4	

Abbreviations: GQS, global quality scale; IQR, interquartile range; mDISCERN, modified DISCERN; PEMAT A/V, patient education materials assessment tool for audiovisual content; VIQI, video information and quality index.

#### mDISCERN

The median mDISCERN score was 2 (IQR: 1–3), depicting low video reliability, but videos created by healthcare professionals scored significantly higher than those produced by patients (2 [IQR: 2–3] vs. 1 [IQR: 0–2]; *P* = 0.001) (Table 3; Figure 2b).

#### VIQI

The overall video information and quality index (VIQI) scores reflected intermediate‐level content, with a median score of 12 (IQR: 10–15). However, videos produced by healthcare professionals received significantly higher scores compared to those created by patients (13 [IQR: 12–17] vs. 9 [IQR: 5–12]; *P* < 0.001). This pattern was consistent across all VIQI subcategories, including information flow (VIQI 1), accuracy of information (VIQI 2), video quality (VIQI 3), and precision (VIQI 4), patients' videos scoring notably lower (all *P* < 0.001; Table 3; Figure 2c).

#### GQS

Similarly, the GQS results showed intermediate usefulness, structure and accessibility of the video content, with an overall median of 3 (IQR: 2.4), but healthcare professional‐created videos had significantly higher scores (median 4 [IQR: 3–4] vs. 2 [IQR: 2–3]; *P* < 0.001) (Table 3; Figure 2d).

### 3.4 Variable correlations

Each tool was correlated with any of the videographic attributes (video length, number of views, comments, likes shares, bookmarks and author's likes) of the included TikTok content. No associations were found between the quality video tools (PEMAT A/V, mDISCERN, VIQI and GQS) and all the videographic attributes (all *P* > 0.05) except for the video length, which was positively correlated with VIQI (*r* = 0.38; *P* < 0.001) and mDISCERN (*r* = 0.24; *P* < 0.001).

## 4 DISCUSSION

This cross‐sectional analysis showed that the overall quality and reliability of TikTok content on PCOS is of low‐intermediate quality. However, several discrepancies occur when distinguishing between healthcare professionals and patients' videos. Healthcare professionals' videos had better understandability than those created by patients, with a marked prevalence of lower quality and partially inaccurate videos by non‐medical users, posing a significant risk of spreading misinformation.

We observed low levels of understandable and actionable content based on our findings. We believe that such issue is related to the fact that both clinicians and patients primarily aimed to raise awareness and highlight macroscopic signs of the condition, particularly hirsutism and menstrual irregularities, while paucity of information regarding the diagnostic processes for PCOS was available.^33^

In contrast, while both healthcare professionals' and patients' videos were poorly actionable, those created by healthcare professionals were closer to being understandable, emphasizing that videos created by patients were lacking in both clarity and practical usefulness.

Previous studies evaluating PCOS‐related content on similar platforms have identified significant issues with misinformation and low‐quality content. A study by Naroji et al., comparing PCOS‐related topics on TikTok, Reddit and Instagram, found a broader trend across social platforms in disseminating incomplete or inaccurate health information.^34^ In a sample of 100 posts, conflicts of interest—such as influencers promoting supplements or health coaching services—were identified in 45% of TikTok content, while higher percentages were observed on Reddit.^34^ On TikTok, weight‐related discussions were the most frequently mentioned topic, whereas ovarian cysts were the least. In contrast, on Instagram, diet was the most common topic, with oral contraceptive pills being the least mentioned. Reddit posts showed the highest engagement on topics related to “symptom management” and “community experiences,” while those focused on “weight management,” “healthcare providers,” and “general questions” garnered the fewest comments.^34^ Therefore, the issue of misinformation on social media is a growing concern. Recent evidence showed that TikTok, with its short‐form video content, is particularly susceptible to disseminating inaccurate health information due to the lack of content verification processes.^35^ Our findings are consistent with those from Ali Baig et al.,^10^ who observed the same high frequency of unverified claims and potentially harmful advice also on YouTube.^1^

Given the unique nature of TikTok's algorithm‐driven and engaging video format, it could make health information more accessible, especially for PCOS‐related health information and similar diseases of adolescent patients. However, the high prevalence of influencer‐driven content, often with conflicts of interest and misinformation, underscores the importance of providing a fact‐checking system to help viewers navigate the social network's content and, at the same time, of counseling young patients to critically evaluate online health information and seeking evidence‐based sources. Therefore, healthcare providers should consider engaging with social media platforms to produce evidence‐based content tailored for a lay audience. Educational programs focused on digital communication skills for healthcare professionals may also be beneficial.

Conversely, regarding chat‐related social media platforms, two studies from China examined the use of WeChat to provide lifestyle advice, reporting a positive contribution.^36^, ^37^ For individuals with PCOS undergoing assisted reproductive technology treatments, advice delivered through WeChat enhanced self‐management abilities, particularly in weight control, and improved oocyte quality.^36^, ^37^ These findings show that, although less direct and engaging, contact with an expert is more appropriate and trustworthy than retrieving information from a short video.

### 4.1 Strengths and limitations

The present study emerged from the objective of evaluating the quality of TikTok videos related to PCOS by employing several validated tools, with the aim of determining whether these short videos could help patients dispel myths and acquire scientifically accurate knowledge. To our knowledge, no prior research has specifically addressed this objective. Our study fills this gap and reveals several noteworthy findings, which we consider to be one of its strengths.

However, there are certain limitations and methodological challenges that should be acknowledged. The video selection process, influenced by TikTok's algorithm and its sensitivity to specific keywords, may have introduced bias, potentially impacting the generalizability of our results. The search terms, algorithm, and selection criteria may have led to an over‐ or underrepresentation of content from different healthcare contexts, professional perspectives, and topic categories. For instance, the search was restricted to the keyword “PCOS,” meaning that relevant videos tagged with other terms (e.g., hirsutism, weight loss, insulin resistance) were excluded from the analysis. Moreover, videos in languages other than English were excluded. Therefore, unrepresented cultural differences might impact the type of content shared, its reception, and the application of such findings in a non‐English context.

Additionally, it is important to note that the TikTok algorithm remains suboptimal in filtering search results based on specific keywords. Of the 300 videos retrieved using the keyword “PCOS,” 107 were considered irrelevant by the authors due to the off‐topic content but were still included in the platform's search results.

## 5 CONCLUSIONS

This analysis of 120 TikTok videos revealed that more than 60% contained partially accurate or uninformative content, with patient‐created videos significantly more likely to include misinformation than healthcare professionals' videos. To date, PCOS‐related TikTok contents have not proven to be a trustworthy source of accurate information in terms of, trustworthiness, correctness, understandability and actionability. The quality and reliability of videos produced by healthcare professionals were generally greater than those made by PCOS‐affected women. It is urged to give women more reliable and accurate content to prevent the spread of untrue myths, wrong therapeutic approaches, and misconceptions about PCOS and to offer higher standards of health education on TikTok and other social media platforms.

## AUTHOR CONTRIBUTIONS

AE and GR conceived and designed the study. CR, AE, GR and LDC contributed to the methods of the study. RMC and CC selected the videos and extracted the data. AE, GR, ASL and PDF analyzed the data. VU, MLV accessed and verified the data. AE and GR wrote the first draft of the manuscript. PDF, MLV, CC, RMC, VU, CR and ASL interpreted the data and contributed to the writing of the final version of the manuscript. All authors agreed with the results and conclusions of the manuscript.

## FUNDING INFORMATION

No funding was obtained for this study.

## CONFLICT OF INTEREST STATEMENT

The authors have no conflicts of interest to declare.

## Data Availability

The data that support the findings of this study are available from the corresponding author upon reasonable request.
